# The endosymbiont of *Epithemia clementina* is specialized for nitrogen fixation within a photosynthetic eukaryote

**DOI:** 10.1093/ismeco/ycae055

**Published:** 2024-04-15

**Authors:** Solène L Y Moulin, Sarah Frail, Thomas Braukmann, Jon Doenier, Melissa Steele-Ogus, Jane C Marks, Matthew M Mills, Ellen Yeh

**Affiliations:** Department of Pathology, Stanford School of Medicine, Stanford, CA 94305, United States; Department of Biochemistry, Stanford School of Medicine, Stanford, CA 94305, United States; Department of Pathology, Stanford School of Medicine, Stanford, CA 94305, United States; Department of Biochemistry, Stanford School of Medicine, Stanford, CA 94305, United States; Department of Biochemistry, Stanford School of Medicine, Stanford, CA 94305, United States; Department of Pathology, Stanford School of Medicine, Stanford, CA 94305, United States; Department of Biological Sciences, Northern Arizona University, Flagstaff, AR 86011, United States; Center for Ecosystem Science and Society, Northern Arizona University, Flagstaff, AZ 86011, United States; Department of Earth System Science, Stanford Doerr School of Sustainability, Stanford, CA 94305, United States; Department of Pathology, Stanford School of Medicine, Stanford, CA 94305, United States; Department of Microbiology & Immunology, Stanford School of Medicine, Stanford, CA 94305, United States; Chan Zuckerberg Biohub, San Francisco, CA 94158, United States

**Keywords:** endosymbiosis, nitrogen fixation, cyanobacteria, diatom

## Abstract

*Epithemia* spp. diatoms contain obligate, nitrogen-fixing endosymbionts, or diazoplasts, derived from cyanobacteria. These algae are a rare example of photosynthetic eukaryotes that have successfully coupled oxygenic photosynthesis with oxygen-sensitive nitrogenase activity. Here, we report a newly-isolated species, *E. clementina*, as a model to investigate endosymbiotic acquisition of nitrogen fixation. We demonstrate that the diazoplast, which has lost photosynthesis, provides fixed nitrogen to the diatom host in exchange for fixed carbon. To identify the metabolic changes associated with this endosymbiotic specialization, we compared the *Epithemia* diazoplast with its close, free-living cyanobacterial relative, *Crocosphaera subtropica*. Unlike *C. subtropica*, in which nitrogenase activity is temporally separated from photosynthesis, we show that nitrogenase activity in the diazoplast is continuous through the day (concurrent with host photosynthesis) and night. Host and diazoplast metabolism are tightly coupled to support nitrogenase activity: Inhibition of photosynthesis abolishes daytime nitrogenase activity, while nighttime nitrogenase activity no longer requires cyanobacterial glycogen storage pathways. Instead, import of host-derived carbohydrates supports nitrogenase activity throughout the day-night cycle. Carbohydrate metabolism is streamlined in the diazoplast compared to *C. subtropica* with retention of the oxidative pentose phosphate pathway and oxidative phosphorylation. Similar to heterocysts, these pathways may be optimized to support nitrogenase activity, providing reducing equivalents and ATP and consuming oxygen. Our results demonstrate that the diazoplast is specialized for endosymbiotic nitrogen fixation. Altogether, we establish a new model for studying endosymbiosis, perform a functional characterization of this diazotroph endosymbiosis, and identify metabolic adaptations for endosymbiotic acquisition of a critical biological function.

## Introduction

Nitrogen comprises 78% of the atmosphere; however, it is present in the form of dinitrogen (N_2_), an inert gas which cannot be directly incorporated into organic molecules. Nitrogen fixation, a critical biological reaction, is the reduction of N_2_ into bioavailable ammonia catalyzed by the enzyme nitrogenase. Biological nitrogen fixation is largely restricted to certain genera of bacteria and archaea. Nitrogen availability determines photosynthetic productivity in both natural environments and agriculture, such that the activity of nitrogen-fixing organisms, or diazotrophs, is a critical source of bioavailable nitrogen for plants and other photosynthetic organisms. To bypass the dependence of food production on biological nitrogen fixation, modern agriculture uses energy from fossil fuels to chemically fix nitrogen and produce nitrogen-rich fertilizer, which is estimated to feed 45% of the world population [1]. However, synthetic fertilizers have significant negative environmental consequences including greenhouse gas emissions, soil degradation, and coastal dead zones (eutrophication) with nitrogen runoff polluting waterways [2–4].

Given the importance of nitrogen for primary production, it may be puzzling why many photosynthetic organisms have not evolved nitrogen fixation function. On one hand, nitrogenase requires 16 ATP molecules and 8 reducing equivalents to fix a single N_2_ molecule, energy that can be supplied by photosynthesis. Thus, coupling nitrogen fixation and photosynthesis in a single cell is energetically advantageous. On the other hand, nitrogenase is extremely sensitive to irreversible inactivation by oxygen which is produced during photosynthesis, such that photosynthesis and nitrogen fixation are biochemically incompatible. Instead, throughout evolution, diverse photosynthetic eukaryotes have coupled these two biologically critical reactions via symbioses with diazotrophic bacteria [5]. The most well-known plant example is legumes which host diazotrophic bacteria in root nodules.

Amongst these diverse symbiotic interactions, a few rare microalgae have acquired diazotrophic cyanobacterial endosymbionts, allowing them to grow in nitrogen-limited environments. *Crocosphaera* spp. cyanobacteria (previously named *Cyanothece*) appear to be a privileged endosymbiotic partner [6]. Several microalgae, the diatoms *Epithemia* spp. and *Climacodium frauenfeldianum* [7] as well as the coccolithophore *Braarudosphaera bigelowii* [8], have permanent endosymbionts closely related to the *Crocosphaera* genus, specifically *Crocosphaera subtropica* (*Cyanothece* sp. ATCC51142). Within their photosynthetic hosts, most of these cyanobacterial endosymbionts have lost their photosynthetic function but retained their ability to fix nitrogen. The remarkable success of *Crocosphaera* cyanobacteria to transition from free-living diazotrophic phototroph to endosymbiotic diazotrophic heterotroph in multiple eukaryotic lineages suggests pre-existing traits that predispose them to endosymbiotic acquisition. For example, *C. subtropica* can grow mixotrophically (i.e. relying on photosynthesis to fix carbon or using carbon substrates from the environment), which may favor its success as an endosymbiotic partner [9, 10].

*Crocosphaera*’s partnership with *Epithemia* diatoms has been particularly successful. Diverse *Epithemia* species containing endosymbionts are widespread in freshwater habitats globally and have recently been found in marine environments [11]. Their remarkable ability to fix both nitrogen and carbon plays a critical ecological role in the food web of aquatic environments [12, 13]. The endosymbionts, first described as “spheroid bodies” within *Epithemia gibba* (formerly *Rhopalodia gibba* [14]), were shown to encode an intact nitrogen fixation (*nif*) gene cluster and demonstrated nitrogenase activity [15–18]. Hereafter, we will refer to these nitrogen-fixing endosymbionts as diazoplasts, combining “diazo-” meaning “relating to the group N_2_” and “-plast” from the Greek *plastos* meaning “formed”. Sequencing of diazoplast genomes from two *Epithemia* species revealed significant genome reduction, including loss of most of the genes involved in photosynthesis, indicating that diazoplasts are dependent on the diatom for fixed carbon [18–20]. Consistent with being obligate endosymbionts, diazoplasts have never been observed outside their diatom, and so far, isolated diazoplasts cannot be grown in laboratory cultures outside the host cells. Diazoplasts are vertically transmitted during cell division and show uniparental inheritance during sexual reproduction, demonstrating robust mechanisms of inheritance [21]. Although *Epithemia* spp. are morphologically diverse, their diazoplasts are believed to derive from a single endosymbiotic event with a cyanobacteria from the *Crocosphaera* genus [22]. Dated at ~35 Mya [23–25], this is the most recent obligate primary endosymbiosis documented to date. Finally, several *Epithemia* strains isolated from environmental samples have been successfully grown in laboratory cultures [26]. Overall, the diazoplasts of *Epithemia* diatoms are an ideal case study for both the acquisition of nitrogen fixation function by a photosynthetic eukaryote and investigating evolution associated with endosymbiotic organellogenesis [19].

Though *Epithemia*’s diazoplasts were described nearly half a century ago, little is known about this functionally unique and ecologically widespread endosymbiotic interaction. So far, the most detailed molecular studies have focused on the extensive genome reduction in the diazoplast pointing toward metabolic dependence on the host cell [18, 19]. While the pattern of reduction and retention in the diazoplast genome suggests new cell biology, such as the energetic coupling of diatom photosynthesis to diazoplast nitrogen fixation, functional studies to support these hypotheses have been limited. Herein, we compare the *Epithemia* diazoplast with a close free-living relative to identify adaptations that occurred in the endosymbiotic transition, i.e., what is the source of energy for nitrogen fixation and how is nitrogenase protected from oxygen evolved during photosynthesis. The diazoplast was investigated in a newly isolated *Epithemia* species, *E. clementina*, while *C. subtropica* served as a proxy for the ancestral free-living cyanobacteria. Our studies reveal key metabolic adaptations accompanying the transition from free-living to endosymbiont that allowed diazoplasts to become specialized for nitrogen fixation within a photosynthetic eukaryote.

## Materials and methods

Details of the Materials and Methods used in this paper, including: Strain isolation, cultivation, and microscopy; Sequencing of the diazoplast genome; Phylogenetic analysis; Metagenomic analysis and assembly; Isotope labeling and NanoSIMS analysis; Transmission Electron Microscopy (TEM); Nitrogenase activity assay; Protein extraction and immunoblot; Gene expression analysis; Clark electrode measurements are provided in SI Appendix, Materials and Methods.

## Results

### *Epithemia clementina* is a new model system to study evolution of diazoplasts

*Epithemia* diatoms are widely distributed in freshwater environments globally, and laboratory cultures have been previously established from isolates collected in the US, Germany, and Japan. Of these cultures, only one *Epithemia* strain has been deposited in a culture collection (*R. gibba*, FD213, UTEX); however, we were unable to establish cultures from this isolate. A previous study has reported *Epithemia* sp. in the Eel river in Northern California [12]. Therefore, to obtain a strain of *Epithemia* for laboratory studies, we collected water samples from a freshwater stream near Butano State Park, CA. The samples were serially diluted over a period of 8 weeks in media depleted of organic carbon and combined nitrogen to isolate diazotrophic phototrophs. Most isolates were dominated by green filamentous cyanobacteria, likely *Nostoc* spp. that are ubiquitous diazotrophic phototrophs. However, a few isolates were visibly brown and consisted of diatoms resembling previously described *Epithemia* spp. that contained endosymbionts. Consistent with their unique ability to fix nitrogen, these diatoms were the only eukaryotic algae that were isolated from passage in nitrogen-free media.

**Figure 1:**
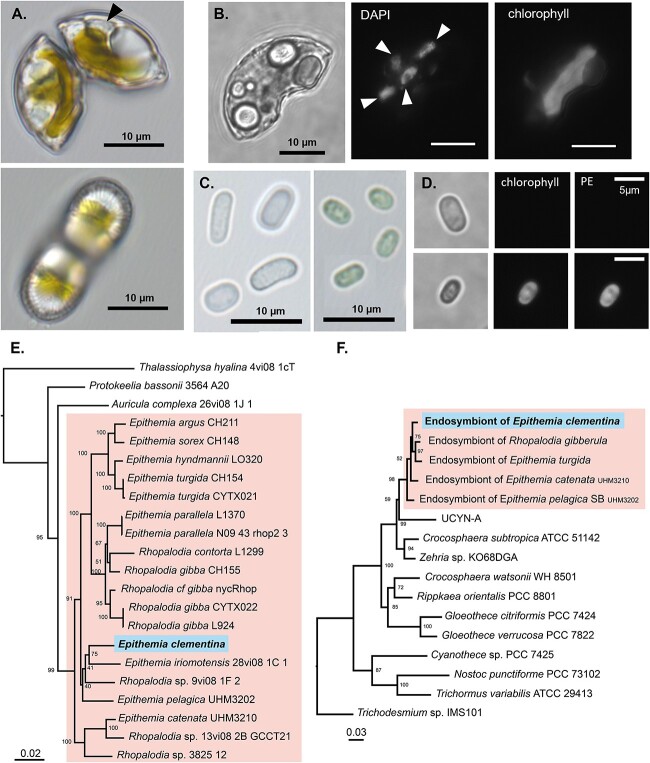
Identification of *Epithemia clementina*. (A) Light microscopy of *E. Clementina* apex (top) and valve/ventral region (bottom) with diazoplast (black arrow). (B) Epifluorescence microscopy of *E. Clementina* comparing brightfield (left), DAPI stain (middle) showing four diazoplasts (white arrows), and chlorophyll fluorescence (right). (C) Light microscopy of isolated endosymbionts released from crushed *E. Clementina* (left), compared with free-living *C. subtropica* (right). (D) Epifluorescence microscopy of isolated endosymbionts (top) and *C. subtropica* (bottom) comparing brightfield (left), chlorophyll fluorescence (middle), and phycoerythrin (PE) fluorescence (right). (E) Multigene phylogeny of *E. Clementina* and related diatoms based on the ribosomal small subunit RNA (18S rRNA), *psbC*, and *rbcL* genes. The highlighted species are from the *Epithemia* genus. (F) Multigene phylogeny of the *E. clementina* endosymbionts and related cyanobacteria, based on the ribosomal small subunit RNA (16SrRNA) and *nifH* genes. The highlighted species correspond to *Epithemia* symbionts. Phylogeny is supported by bootstrap 921 values, and phylogeny scales are in units of nucleotide substitutions per site. Accession numbers for all sequences are provided in source data file (Table S4).

Under light microscopy, the isolated alga was ~20 μm long and resembled an orange wedge (Fig. 1A). Because morphologic and phylogenetic characterization (detailed below) suggest it is a new species, it was named *Epithemia clementina*. Like other members of its genus, it is a raphid pennate diatom possessing lunate, planar valve faces, and prominent internal costae. It has an asymmetrical growth of its girdle bands and shows the presence of marginal raphes on its concave side. Morphologically, the shape and pattern associated with the valve does not match other described *Epithemia* species (Fig. S1A). Similar to other *Epithemia* diatoms, *E. clementina* contains endosymbionts appearing as spheroid bodies within the diatom both under light microscopy and by fluorescence microscopy after staining for nucleic acids (Fig. 1A and B). Nucleic acid staining also revealed the presence of bacteria attached to the frustule of *E. clementina.* Consistent with previous characterization of diazoplasts, the isolated endosymbionts were colorless in light microscopy and did not have any signal in fluorescence microscopy, indicating a lack of photosynthetic pigments (Fig. 1C and D, Fig. S1B).

The morphologic classification was supported by molecular phylogeny. Phylogenetic analysis based on three conserved genes from diatoms confirmed that this previously undescribed species is a member of the *Epithemia* genus, most closely related to *Epithemia iriomotensis* (Fig. 1E) [14]. To identify the origin of the endosymbiont, we also performed a phylogenetic analysis of two conserved cyanobacterial genes which showed that *E. clementina* endosymbiont is of the same origin as endosymbionts previously found in *Epithemia* spp. (Fig. 1F) [22]. The diazoplast genome was assembled with a hybrid assembly strategy using Nanopore long-read and Illumina short-read sequencing of a metagenomic sample of our lab isolate containing *E. clementina* and associated bacteria. The genome confirmed that the endosymbiont was indeed a diazoplast, containing an intact *nif* cluster as observed in other *Epithemiaceae* [18, 19]. Having observed bacteria closely associated with *E. clementina* in the monoalgal cultures that could not be removed by dilution, detergent, or antibiotic treatment, we took advantage of our metagenomic sequencing data to characterize the bacterial community of *E. clementina*. 62 metagenome-assembled genomes (MAGs) from taxonomically diverse bacteria were obtained in addition to the diazoplast genome (Fig. S2, Table S1, NCBI assembly GCA_029919255.1). None of the bacteria were assigned as phototrophs, though the presence of *nif* genes was detected in 2 MAGs related to *Methyloversatilis* sp. RAC08 (~90% identity) and *Hyphomicrobium* sp. DMF-1 (~75–80% identity). Based on sequencing depth, the diazoplast was the most abundant bacterial genome and accounted for 19.2% of bacterial genomes detected, compared to <0.3% for the two *nif*-containing MAGs, suggesting that it is the primary source of nitrogenase activity in our cultures.

### The diazoplast transfers fixed nitrogen to diatom host in exchange for fixed carbon

Since diazoplasts can fix nitrogen but have lost photosynthesis, it has been presumed that diazoplasts transfer fixed nitrogen to the diatom host, which in turn provides fixed carbon to the diazoplast to fuel nitrogen fixation, as shown in other diazotroph endosymbioses [27, 28]. However, the metabolic exchange underlying this endosymbiosis has not been demonstrated. To spatially monitor the fixation of both nitrogen gas and inorganic carbon and the transfer of assimilated products, established *E. clementina* cultures were incubated with ^15^N_2_-enriched media supplemented with ^13^C-bicarbonate for 12 h in the light or 24 h corresponding to a full day/night cycle and imaged by nanoscale secondary ion mass spectrometry (nanoSIMS). Nitrogen was measured by the formation of CN^−^, while carbon was measured by CC^−^ ions.

**Figure 2:**
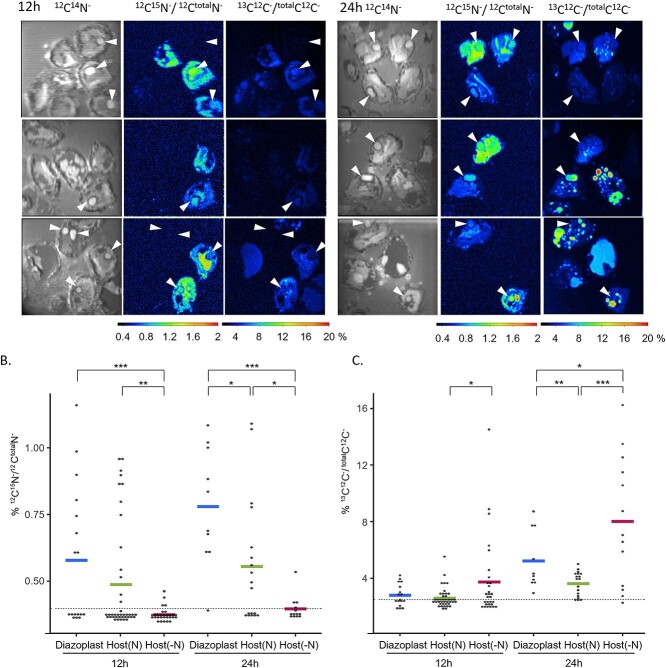
Nitrogen and carbon fixation and transfer between diazoplast and host diatom assessed by nanoSIMS. (A) Representative images of cells labeled with ^15^N_2_ and H^13^CO_3_^−^ after 12 h (left three panels) and 24 h (right three panels), showing raw counts for nitrogen (first column), ^15^N atom% (second column), and ^13^C atom% (third column). Arrows indicate diazoplasts. (B) ^15^N atom% and (C) ^13^C atom% for each region of interest. Each cell was separated into three distinct compartments, the diazoplast, host(N) and host(-N). Each measurement is represented by a single point, and colored bars correspond to averages. The dashed line corresponds to the natural abundance observed in unlabeled samples (average atom% + 3*SD). *, **, and *** indicate that treatments are significantly different with a *P*-value <.05, .005, and .0005 respectively.

We first observed incorporation of ^15^N_2_. When *Epithemia* cells were analyzed for ^12^C^14^N^−^ abundance, the diazoplast and two distinct host fractions were identified (Figs 2A and S3): Spherical compartments which showed the highest ^12^C^14^N^−^ counts likely corresponded to diazoplasts (indicated by arrowheads). The host diatom consisted of nitrogen-containing cell areas with above-background ^12^C^14^N^−^ counts (“host(N)”) likely including the nucleus, chloroplast, mitochondria, and cytoplasm in contrast to nitrogen-depleted compartments with background ^12^C^14^N^−^ counts but high ^12^C_2_^−^ counts corresponding to possible lipid droplets or vacuoles (“host(-N)”). (Of note, ^32^S counts were highly correlated with ^12^C^14^N^−^ counts in diazoplast and host cytoplasmic compartments, indicating that the ^12^C^14^N^−^ counts reflected protein abundance (Fig. S3).) The ^15^N atom percent, equal to ^12^C^15^N^−^/^12^C^total^N^−^ counts, was calculated for each compartment separately: diazoplast, host(N), and host(-N) (Fig. 2B). In control samples incubated with atmospheric N_2_, the average ^15^N atom% was 0.377% ±0.012 across compartments and corresponded to the natural abundance of ^15^N. In ^15^N_2_-labeled samples, both diazoplast and host(N) fractions showed enrichment for ^15^N at 12 and 24 h as detected by ^15^N atom% above natural abundance, attributable to fixation of ^15^N_2_ in the diazoplast and transfer of assimilated products to the host(N) compartments (Fig. 2A and B). To estimate the relative amount of fixed nitrogen in diazoplast versus host, we compared the ^15^N atom% of diazoplast and host(N) fractions for the subset of cells for which both measurements were available (Fig. S4A). Linear regression showed that the host ^15^N enrichment was 0.9 that of the diazoplast (R^2^ = 0.97). With an estimated diazoplast/host(N) volume ratio of 6%, the nearly equivalent ^15^N enrichment in the diazoplast and host suggests that ~93% of nitrogen fixed by the diazoplast during the experiment was transferred to the host. Taken together, these results are consistent with fixation of N_2_ in the diazoplast followed by transfer of most of the fixed nitrogen to the diatom host.

Several other findings were notable. First, there was variability in the nitrogenase activity of individual cells with some cells showing no activity (Fig. 2A and B), an observation very similar to patterns shown in *C. subtropica* populations [29]. Second, the fraction of cells containing actively-fixing diazoplasts was 70% after 12 h of light and 90% after the 24 h full day-night cycle, demonstrating that *E. clementina* diazoplast performs nitrogen fixation during both day and night periods (Fig. 2B). Third, none of the organic material outside the *E. clementina* cells showed ^15^N enrichment, supporting the fact that the bacterial community co-cultured with *E. clementina* does not provide a significant amount of fixed nitrogen (Fig. 2A). Lastly, though nitrogen-rich cyanophycin granules were observed in *C. subtropica* [29], we did not observe the formation of ^15^N-enriched granules in the diazoplast suggesting that it does not store fixed nitrogen. Consistent with this result, the genes responsible for cyanophycin metabolism are missing in the genome of the diazoplast (Table S2).

We next followed the incorporation of ^13^C-bicarbonate in *Epithemia* cells. Based on unlabeled control samples, the natural abundance of ^13^C was determined to be 1.74% ±0.5. In samples supplemented with ^13^C-bicarbonate, diazoplast and both host fractions showed ^13^C atom% (^13^C^12^C^−^/^total^C^12^C^−^ counts) significantly higher than natural abundance at 24 h (Fig. 2A). The enrichment of ^13^C in the diazoplast is consistent with the transfer of carbon fixed in the diatom chloroplast present in the host(N) fraction to the diazoplast (Fig. 2C). In addition, the majority of fixed carbon accumulated in the host(-N) fraction, suggesting that it is indeed a storage compartment. Surprisingly, though carbon fixation is believed to occur during the light period, we noted that ^13^C enrichment in *Epithemia* cells was very low after the 12 h light period and significantly greater after a full 24 h light–dark cycle (Fig. 2C). However, the sample preparation used for nanoSIMS includes washing and dehydration steps that likely flush away simple sugars produced by photosynthesis during the day. To rule out a potential nighttime carbon fixation as known in CAM plants, we quantified ^13^C bicarbonate incorporation in the total biomass using isotope ratio mass spectrometry (IRMS). This method measures the bulk isotopic ratio directly in dried organic samples therefore minimizing loss of metabolites. After a 12 h day or night ^13^C bicarbonate incubations, δ^13^C reached 2837‰ ±850 and 16‰ ±5 respectively while ^12^C bicarbonate controls remained at −19‰ ±2 (Fig. S5). These results are consistent with the carbon fixation reactions of photosynthesis occurring only during the light period.

Altogether these results suggest that, during the light period, the products of photosynthesis, simple sugars, are not directly converted to storage forms that would be retained during fixation and wash steps of sample preparation for the nanoSIMS. Instead, complex carbohydrates and/or lipids may accumulate primarily during the dark period in the host(-N) compartments corresponding to possible lipid droplets or vacuoles. Finally, since nanoSIMS allows observation of heterogeneity in the population, we compared ^13^C and ^15^N enrichment within individual *Epithemia* cells. The majority of cells performed both carbon and nitrogen fixation, while only a few cells had clear carbon fixation with no nitrogenase activity and vice versa (Fig. S4B).

### New daytime nitrogenase activity in the diazoplast is tightly coupled to host photosynthesis

**Figure 3:**
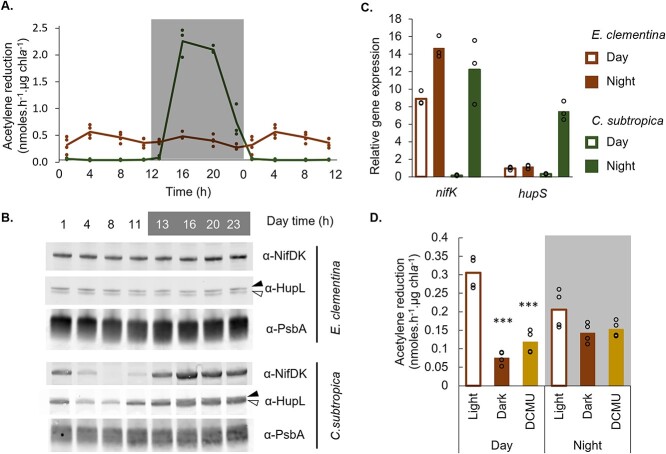
Diel pattern of nitrogen fixation in *E. clementina* and *C. subtropica* ATCC 51142 and coupling with host photosynthesis. (A) Nitrogenase activity assessed by acetylene reduction assay (ARA) over a diel cycle in *E. Clementina* (brown, top at time zero) and *C. subtropica* ATCC 51142 (green, bottom at time zero) normalized by chlorophyll content. Timepoints (1, 4, 8 and 11 h) were duplicated for better visualization of the cycle. (B) Corresponding immunoblot for nitrogenase (NifDK) and hydrogenase uptake (HupL). Both apo-HupL (black arrow) and mature HupL protein (white arrow) were detected. (C) Relative gene expression of *nifK* and *hupS* in *E. Clementina* (brown) and *C. subtropica* (green) during early day (open bars) and early night (solid bars). (D) Nitrogenase activity assessed by ARA at 4 h into day or night in *E. clementina*, comparing photosynthetic (light) and non-photosynthetic conditions (dark or DCMU treatment). Mean values are plotted with individual biological replicates indicated by data points. *** indicate that treatment is significantly different from control condition with a *P*-value <.0005.

Having verified the metabolic exchange underlying the endosymbiosis, we sought to identify metabolic changes in the diazoplast associated with its endosymbiotic specialization. In addition to establishing laboratory cultures of *E. clementina* to study its diazoplast, we obtained cultures of *C. subtropica*, which is closely related to the diazoplast (Fig. 1F), to serve as a proxy for the free-living ancestor of the diazoplast. We first compared the temporal regulation of nitrogenase activity in free-living *C. subtropica* and *E. clementina*’s diazoplast. Nitrogenase is sensitive to deactivation by oxygen; as a result, mechanisms to separate nitrogenase activity and oxygenic photosynthesis either spatially or temporally have evolved in cyanobacteria like *C. subtropica* that perform both. Studies of *C. subtropica* demonstrated tight transcriptional and post-translational regulation of nitrogenase, such that nitrogen fixation only occurs at night when photosynthesis is inactive [30–33]. Using an acetylene reduction assay (ARA), we confirmed that the nitrogenase activity of *C. subtropica* was restricted to nighttime with a strong diel pattern that peaked around 4 h into the night (Fig. 3A) [32, 34, 35]. In contrast to *C. subtropica*, the diazoplast is no longer photosynthetic with photosynthesis occurring in a separate cellular compartment, the host chloroplast. Previous studies of other *Epithemia* spp. have demonstrated daytime nitrogenase activity concurrent with photosynthesis, which we also observed in nanoSIMS analyses of *E. clementina* cells, indicating a remodeling of the temporal regulation [11, 15, 16]. Consistent with these results and in contrast to free-living *C. subtropica*, *E. clementina* showed nitrogenase activity throughout the day-night cycle with no clear diel pattern (Fig. 3A).

The loss of temporal regulation of diazoplast nitrogenase activity was reflected in changes in the expression of *nif* and associated genes. In *C. subtropica*, immunoblotting for the NifDK subunits of nitrogenase showed a diel pattern matching the nitrogenase activity, while in *E. clementina*, NifDK protein abundance was stable throughout the day-night cycle (Figs 3B and S6). We also assessed the abundance of HupL, the large subunit of the uptake hydrogenase which recycles hydrogen produced by nitrogenase and is often co-expressed with *nif* genes [36]. HupL protein abundance also showed diel variation peaking at night in *C. subtropica*, while stable protein levels were detected in the diazoplast (Fig. 3B). We also performed RT-qPCR to detect transcript levels of the NifK subunit of nitrogenase and HupS, the small subunit of the uptake hydrogenase. Both *nifK* and *hupS* showed tightly regulated nighttime expression in *C. subtropica* while the diazoplast showed constitutive *nifK* and *hupS* expression (Fig. 3C).

New daytime nitrogenase activity in the diazoplast, concurrent with host photosynthesis, permits energetic coupling of nitrogen fixation with photosynthesis that was not previously possible in *C. subtropica*. To investigate the coupling between host photosynthesis and diazoplast nitrogenase activity, ARAs were performed under light, dark, and light + an inhibitor of photosystem II, 3-(3,4-dichlorophenyl)-1,1-dimethylurea (DCMU). During the day, inhibition of the host photosynthesis by dark condition or DCMU treatment reduced nitrogenase activity by 60% (Fig. 3D). At night, host photosynthesis by addition of light provided minimal benefit for nitrogenase activity (Fig. 3D). These results suggest that photosynthates are directly shuttled from the host chloroplast to the diazoplast during the day, while nighttime nitrogenase activity relies on carbohydrate storage as expected. The ability of daytime nitrogen fixation, concurrent with photosynthesis, permits a direct use of host photosynthates without reliance on carbohydrate storage. Moreover, in both conditions, blocking oxygen production with DCMU did not increase nitrogenase activity, indicating a mechanism to prevent inactivation of nitrogenase by the oxygen produced by host photosynthesis.

### Nighttime nitrogen fixation is no longer dependent on diazoplast glycogen storage

**Figure 4:**
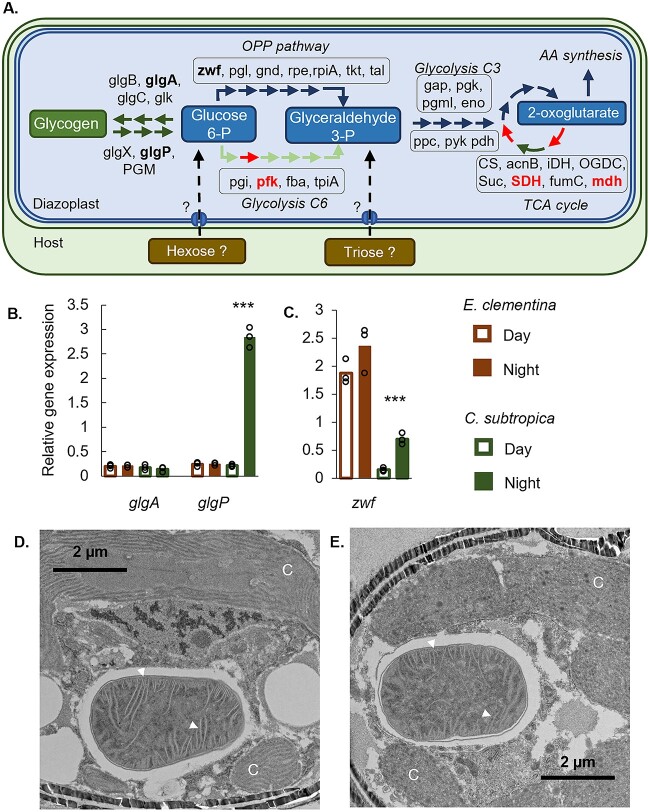
Carbohydrate metabolism associated with nitrogen fixation in the diazoplast. (A) Annotated carbohydrate metabolism pathways in the diazoplast. Proteins only present in *C. subtropica* and absent in diazoplast’s genome are indicated in red. See Table S2 for corresponding gene ID numbers. (B-C) relative gene expression of representative genes in glycogen biosynthesis (*glgA*), glycogen degradation (*glgP*), and OPP (*zwf*) pathways in *E. clementina* (brown) and *C. subtropica* (green) during early day (open bars) and early night (solid bars). Mean values are plotted with individual biological replicates indicated by data points; *** indicate that treatment is significantly different from control condition with a *P*-value <.0005. (D-E) representative TEM images of *E. clementina* showing thylakoids in the diazoplast (arrows) and host chloroplasts (C) at the end of day (D) or night (E).

To determine the source of energy fueling nighttime nitrogen fixation in the diazoplast, we assessed its glycogen metabolism. In cyanobacteria, glycogen is commonly used to store excess photosynthate produced during the day [37]. Global transcriptomic analysis of the diel cycle *in C. subtropica* confirmed: (i) upregulation of photosynthesis and glycogen biosynthesis genes during the day and (ii) upregulation at night of nitrogen fixation, glycogen degradation, and glucose catabolism pathways [38]. These observations pointed to a model whereby *C. subtropica* converts excess glucose to glycogen during the day, which is then catabolized at night to provide ATP and reducing equivalents for nitrogenase activity [32, 35]. Despite gene reduction in glycogen metabolism pathways, the diazoplast genome encodes at least one copy of each gene required for glycogen biosynthesis and degradation, suggesting these pathways are functional (Fig. 4A, Table S2). If the diazoplast accumulates glycogen as an intermediate storage form of host photosynthate to fuel nighttime nitrogen fixation, glycogen biosynthesis and degradation pathways are expected to be regulated in a diel pattern. Therefore, we measured the daytime and nighttime expression of genes related to glycogen biosynthesis (glycogen synthase, *glgA*) and glycogen degradation (glycogen phosphorylase, *glgP*) by RT-qPCR. No diel variation of *glgA* expression was observed in either *E. clementina* or *C. subtropica* (Fig. 4B). Expression of *glgP* was upregulated by over 10-fold at night compared to the day in *C. subtropica*, consistent with the catabolism of stored glycogen to provide energy for nighttime nitrogen fixation in this organism. In contrast, no diel variation of *glgP* was seen in the diazoplast. In fact, relative *glgP* expression was consistently low in the diazoplast, despite high *nifK* expression, and comparable to daytime *glgP* relative expression levels in *C. subtropica* when the glycogen degradation pathway is effectively inactive (Fig. 4B).

The status of glycogen storage in the diazoplast was also visualized by microscopy. In *C. subtropica*, glycogen has been shown to accumulate during the day in granules observed by electron microscopy and nanoSIMS [29, 35, 39]. This cycling of the glycogen synthesis and degradation was even observed in heterotrophic condition when *C. subtropica* cultures were kept in the dark and using glycerol as their only source of carbon [9, 40]. Moreover, nitrogenase activity was impaired when glycogen granules were experimentally depleted by dark incubation during the preceding day period [35]. However, previously published images of *Epithemia* spp. did not show similar granules [15, 41] nor were ^13^C-enriched granules detected in *E. clementina* diazoplasts by nanoSIMS (Fig. 2A). Finally, when we imaged diazoplasts of *E. clementina* by EM, no glycogen granules were apparent at the end of the daytime nor at the end of the night (Fig*.* 4D and E). Altogether, these results indicate that glycogen synthesized in the diazoplast is not the source of energy for nitrogenase even during nighttime. Instead, glycogen pathways may be retained in the diazoplast for other purposes or under different conditions. Since other pathways for polysaccharide biosynthesis were not detected in the diazoplast genome, nighttime nitrogen fixation in the diazoplast most likely requires mobilization of host storage carbohydrates. If so, carbohydrate utilization in the diazoplast demonstrates tight coupling between host and diazoplast metabolism beyond that expected to support daytime nitrogen fixation.

### Streamlined carbohydrate catabolism in the diazoplast favors a reducing environment

Biological nitrogen fixation is an energy-intensive reaction; therefore, the metabolism of diazotrophs must have the ability to supply large quantities of both ATP and reducing equivalents to the nitrogenase. After examining the sources of carbohydrate for daytime and nighttime nitrogen fixation in the diazoplast, we turned our attention to catabolism in the diazoplast to convert host-derived carbohydrates into ATP and reducing equivalents. *C. subtropica* has been shown to rely on glycolysis, the oxidative pentose phosphate (OPP) pathway, and the tricarboxylic acid (TCA) cycle for carbohydrate catabolism [38]. However, of these, the OPP pathway is the only complete pathway in diazoplasts, since many of the TCA cycle genes and the gene encoding the “gatekeeper” enzyme of glycolysis, phosphofructokinase (PFK), are missing from diazoplast genomes of *E. clementina*, *Epithemia turgida, and Rhopalodia gibberula* [18] (Fig. 4A*,*Table S2). In heterocysts, specialized nitrogen-fixing cells found in filamentous cyanobacteria, nitrogenase activity was shown to be highly dependent on glucose-6-phosphate dehydrogenase (G6PD), a key enzyme of the OPP pathway [42, 43]. To investigate the importance of the OPP pathway in diazoplast metabolism, we compared expression of the gene encoding G6PD (*zwf*) between the diazoplast and *C. subtropica*. The relative expression level of *zwf* (normalized to housekeeping genes) in *E. clementina* was 2.5-3x higher than that of *C. subtropica* under nitrogen-fixing conditions, despite comparable *nifK* expression (Figs 4C and 3C). Consistent with the loss of temporal regulation of nitrogen fixation, *zwf* expression did not exhibit diel variation in the diazoplast. Combined with the loss of key enzymes in glycolysis and TCA pathways, the increase of *zwf* expression suggests that OPP is the main pathway for carbohydrate catabolism in the diazoplast (Fig. 4A). Because G6PD utilizes glucose-6-phosphate as its substrate, the dependence on the OPP pathway in the diazoplast indicates that at least one host-derived carbohydrate is likely a hexose or disaccharide.

In heterocysts, dependence on the OPP pathway was proposed to maximize production of reducing equivalents for oxidative phosphorylation that both generates ATP and consumes oxygen. High levels of oxidative phosphorylation may in turn be important for protecting nitrogenase from inactivation by oxygen produced during photosynthesis. Diazoplasts have retained complete pathways for respiratory electron transport and oxidative phosphorylation [18]. As thylakoid membranes are the site of both photosynthetic and respiratory electron transport complexes in cyanobacteria, we performed electron microscopy to visualize diazoplast internal membranes. Despite the loss of photosystems, we observed the presence of thylakoid membranes in the *E. clementina* diazoplast similar to those observed in *C. subtropica* (Fig. 4D and E). Thylakoids have also been observed in diazoplasts of other *Epithemia* spp. [15, 41]. In absence of photosynthetic electron transport, these membranes likely were retained in diazoplasts to perform respiratory electron transport and oxidative phosphorylation. Supporting the link between high respiratory and nitrogenase activity, in *C. subtropica*, we found that the respiration rate was higher in nitrogen-fixing, nitrogen-depleted condition than when nitrogenase activity is suppressed via ammonia supplementation (Fig. S7) [10]. Unfortunately, similar experiments could not be conducted to measure respiration in *E. clementina* due to the inability to distinguish the respiratory activity of the diazoplast, host diatom, and associated bacteria or purify functional diazoplasts. Overall, carbon catabolism in the diazoplast is streamlined to use the OPP pathway and electron transport/oxidative phosphorylation to convert host-derived carbohydrates to supply energy and generate a microoxic environment for nitrogenase.

## Discussion

Metabolic exchange is often the underlying currency of symbiotic interactions and a strong driver of evolution. Our results demonstrate that the acquisition of nitrogen fixation by *Epithemia* diatoms has led to global metabolic rearrangements in the diazoplast. Whereas *Crocosphaera* cyanobacteria, quintessential generalists, are free-living phototrophs that perform nitrogen fixation exclusively at night, the diazoplast has evolved into a nitrogen-fixing specialist and obligate heterotroph endosymbiont. In reviewing our key findings below, we discuss potential mechanisms for these metabolic changes and how these changes may be tailored to support nitrogenase activity. Highlighting the importance of *E. clementina* as a new model system, the detailed view of metabolic integration described in this unicellular diazotrophic symbiosis has yet to be documented in similar models which lack established laboratory cultures.

### Mechanisms enabling daytime nitrogen fixation

The first notable change we observed was daytime nitrogen fixation in the diazoplast. We observed continuous nitrogen fixation in *E. clementina*, while recently discovered marine *Epithemia* species, *E. pelagica* and *E. catenata*, showed nitrogenase activity over the daytime with a hiatus of activity at night, a reversed day-night pattern compared to *C. subtropica* [11]. This lax temporal regulation is in stark contrast to *C. subtropica*, in which 30% of genes, mostly associated with photosynthesis and nitrogen fixation, show circadian expression [38, 44–46]. Another *Crocosphaera*-related endosymbiosis, UCYN-A, also shows altered temporal regulation of nitrogen fixation with expression of *nif* genes in the daytime [47–50]. UCYN-A has retained photosystem I which may participate in regenerating reduced ferredoxin but, like *Epithemia* diazoplasts, has lost photosystem II necessary for oxygenic photosynthesis. In both endosymbioses, the spatial separation of oxygenic photosynthesis in the host chloroplast may have removed strong selection for temporal regulation of nitrogenase. Cyanobacterial circadian clock relies on three *kai* genes and *C. subtropica* has two copies of each gene while the diazoplast retained one copy of each (Table S2) [51]. It is not clear how the loss of redundancy would affect the circadian rhythm. Alternatively, the loss of redox signals resulting from photosynthesis that synchronizes light and dark periods may weaken the circadian cycle. Finally, we hypothesize that the incomplete TCA cycle in the diazoplast may lead to accumulation of 2-oxoglutarate, which is both a substrate for GS-GOGAT-mediated ammonia assimilation and a signaling metabolite that binds to NtcA and PII, regulatory factors upstream of CnfR, the master regulator of *nif* expression that senses carbon/nitrogen balance [52–56]. If *nif* gene regulation is conserved in diazoplasts, as suggested by the retention of genes encoding NtcA, PII, and CnfR in all diazoplast genomes sequenced so far (Table S2), the accumulation of 2-oxoglutarate would signal a high C:N ratio in the diazoplast, a metabolic state that activates nitrogen fixation and may contribute to the temporal deregulation of nitrogen fixation. Regardless of the mechanism, the consistent shift toward daytime nitrogen fixation in *Crocosphaera*-derived endosymbioses indicates a benefit of direct usage of photosynthates to fuel nitrogen fixation. The cyanobacterium *Trichodesmium*, which also performs nitrogen fixation concurrent with oxygenic photosynthesis, has been proposed to benefit from the absence of glycogen buildup during the day to maintain buoyancy in the water column [57, 58]. In *Trichodesmium*, the mechanism by which nitrogenase is protected from the oxygen produced by photosynthesis is not known.

### What host-provided carbohydrate(s) is utilized by the diazoplast?

We observed that daytime nitrogen fixation was dependent on host photosynthesis, while night time nitrogen fixation was no longer associated with diazoplast glycogen storage and degradation, suggesting the diazoplast relies entirely on carbohydrates imported from the host. Though photosynthetic, some cyanobacteria have been observed to grow mixotrophically on exogenous carbons, usually glucose, fructose, and/or sucrose. Two transporters, GlcP and FrtABC, have been associated with glucose and fructose uptake respectively, in cyanobacteria. However, orthologs of these transporters are not detected in *C. subtropica* nor diazoplast genomes. Similarly, utilization of the disaccharide sucrose requires an invertase or a sucrose synthase (known to catalyze reversible reactions) to hydrolyze it into glucose and fructose, but neither has been detected in *C. subtropica* or diazoplast genomes. Instead, *C. subtropica* shows robust growth on glycerol as a carbon source and utilizes glucose after a prolonged period of adaptation [9, 10]. The transporters that allow *C. subtropica* to readily uptake glycerol and adapt to glucose uptake are unknown, though potential genes annotated as sugar ABC transporters are identified in the genomes of *C. subtropica*, *R. orientalis*, and three diazoplasts. More detailed investigations of mixotrophic growth in *C. subtropica* will be needed to determine whether pre-existing transporter gene(s) were utilized to access host carbohydrates. An alternative model is that the diazoplast acquired the ability to uptake host carbohydrate(s), since we detected genes encoding possible transporters in all diazoplast genomes that were absent from free-living *Crocosphaera* genomes. Further investigation is needed, but these could be exciting transporter candidates. We also considered the possibility that, instead of sugar compounds, ATP and NADPH could be directly imported into the diazoplast. However, none of the transporters known in mitochondria or chloroplasts were identified in diazoplast genomes, and the dependence on the OPP pathway suggests the import of hexose or disaccharide from the host.

### The OPP pathway and oxidative phosphorylation provide reducing equivalents, ATP, and protection from oxygen for nitrogenase

The third change we noted was an incomplete set of glycolysis and TCA cycle genes, likely dependence on an upregulated OPP pathway both at night and during the day for carbohydrate catabolism, and retention of thylakoids in the absence of photosystems. While multiple enzymes in the TCA cycle were missing such that it was unlikely to be reconstituted, only PFK, the enzyme responsible for the first committed step of glycolysis, is absent from the diazoplast genome compared to *C. subtropica*. The absence of PFK is relatively common in cyanobacteria [59, 60]; however, we also consider the possibility that the PFK gene has transferred to the diatom nucleus and is imported. Compared to glycolysis, the OPP pathway favors production of NADPH at the expense of ATP production. NADPH can be used to generate reduced ferredoxins required by nitrogenase via the ferredoxin-NADP^+^ reductase which is retained in diazoplasts. Similarly, UCYN-A has likely retained photosystem I to perform ferredoxin reduction [61, 62]. Reducing equivalents can also enter the respiratory electron transport chain coupled to oxidative phosphorylation, occurring on thylakoid membranes retained in the diazoplast [15, 41]. We propose that, in the diazoplast, oxidative phosphorylation not only provides the large amounts of ATP needed for nitrogenase but also consumes oxygen as the terminal electron acceptor contributing to a microoxic environment for nitrogenase activity. Like *C. subtropica*, all diazoplasts contain two complete sets of genes encoding cytochrome terminal oxidases including a cbb3-type known to have higher affinity for oxygen [63]. Consistent with this model, heterocysts are dependent on the OPP pathway for their nitrogenase activity, and genes related to oxidative phosphorylation are upregulated at the transition from day to night in *C. subtropica* [38, 42, 43].

### Implications for engineering nitrogen-fixing crop plants

Crop plants, particularly cereals, with the ability to fix nitrogen are the holy grail for sustainable agriculture. However, so far, attempts to transfer the genes for biological nitrogen fixation from bacteria into plants have met many challenges, including the large number of genes required, the complexity of nitrogenase assembly, the high cellular energy requirements, and exquisite sensitivity to oxygen [64–68]. Ironically, one of the current strategy relies on targeting nitrogenase to the mitochondria where high respiratory rates provide energy and protects it from inactivation [69–74]. Another strategy is the engineering of root nodule symbioses in non-legume crop species with a major hurdle being the specific recruitment of facultative diazotrophic bacteria from soil and de novo formation of nodules, a specialized organ, to house them [75]. We propose a third strategy: an organelle-like compartment specialized for nitrogen fixation inspired by unicellular diazotroph endosymbioses such as *Epithemia*’s diazoplast and UCYN-A. Although, where the diazoplast is on the evolutionary path between endosymbiont and organelle is unknown, it serves as a ready-made nitrogen-fixing factory, expressing all the genes required, properly regulated, and housed inside a protected membrane compartment. Given the advantages of nitrogen fixation capability, the absence of such an organelle is a mystery but may be an accident of evolutionary timing or a result of incomplete documentation of eukaryotic diversity [76, 77]. Regardless of the status of the diazotroph endosymbiosis in nature, increasing knowledge of these interactions and new genetic tools open the opportunity to revisit this strategy in the laboratory. We propose that *Epithemia* diazoplasts could serve as a blueprint for engineering nitrogen-fixing organelles in plants. The metabolic adaptations we have observed in the diazoplast, for example, offers several options for genetic engineering of a suitable bacterial partner. We are currently unaware of the extent of the diatom host’s contribution to the endosymbiotic interaction, whether solely in the form of metabolites or also via imported gene products. However, analysis of *Epithemia* genomes is underway in our laboratory and will provide insight into gene transfers and imported gene products, if any. Altogether, our investigations of this native endosymbiotic interaction will be a powerful tool for bioengineering.

## Supplementary Material

ISMEcomm-supplementals-submitted_ycae055

TableS1-MAG_summary_ycae055

TableS2_NCBI-updated_ycae055

TableS4-phylogeny-corrected_ycae055

## Data Availability

All data generated or analyzed during this study are included in this published article, its supplementary information files, and NCBI database.
