# Supplementary material for: The endosymbiont of Epithemia clementina is specialized for nitrogen fixation within a photosynthetic eukaryote
Source: ISME Commun. 2024 Apr 15;4(1):ycae055. doi: 10.1093/ismeco/ycae055 (PMC11070190; doi:10.1093/ismeco/ycae055)
Supplement: ISMEcomm-supplementals-submitted_ycae055 [file ismecomm-supplementals-submitted_ycae055.pdf]

Supplemental Appendix

This PDF file includes:

Materials and Methods

Figures S1 to S7

Tables S1 to S5

Materials and Methods

Strain isolation, cultivation, and microscopy

Environmental samples originated from Gazos Creek near Butano State Park, CA, USA and were taken during fall 2020. *E. clementina* was isolated by successive dilution in nitrogen-depleted media, Csi-N (Table S3), under 10 µmole photon.m^{-2}.s^{-1} of white light at 20°C as previously described (1) and were maintained in the same condition. After subculturing for 8 weeks, we obtained many monoalgal isolates, some containing filamentous cyanobacteria and some containing *Epithemia* diatoms. Cultures, were started from single cell isolate of *E. clementina*, and formed a biofilm on the surface of the flaks. Cultures were monoalgal but not axenic. For morphology-based species characterization, we compared the isolate with several *Epithemia* species that were closely related to it by phylogeny, including *E. musculus, E. iriomotensis, E. Agharkarii, E. gibberula*.

Crocosphaera suptropica (*Cyanothece* ATCC51142) was kindly provided by Dr. Jonathan Zehr and maintained at 30°C under day-night cycle (12 h/12 h – 30 µmole photon.m^{-2}.s^{-1}/dark) in artificial sea water ASP2 (2) with constant bubbling of humidified ambient air.

Observation of diazoplasts by DNA stain was performed after fixation for 10 min with 4% paraformaldehyde followed by incubation for 30 min with 1µg/mL DAPI. Fluorescence microscopy was performed with a CY5 and TRITC filter for chlorophyll and phycoerythrin respectively. Diazoplast were

isolated for microscopy from the diatom cells by pressing on the coverslip to crack the diatom open and release to diazoplast.

Sequencing of the diazoplast genome

Epithemia clementina gDNA was extracted using Qiagen DNeasy Plant Pro Kit [69206]. DNA fragments were prepped for Illumina sequencing with NEBNext Ultra II FS DNA Library Prep Kit for Illumina [E7805S]. Sequencing was performed on an Illumina NextSeq 2000 P3 paired end 150bp. Sequencing reads were trimmed, quality filtered with fastp (3) (--qualified_quality_phred 20 –unqualified_percent_limit 20), assembled with SPAades on default settings (4) and quality was assessed with FastQC (5).

For Nanopore sequencing, gDNA was isolated with circulomics nanobind plant nuclei DNA kit. Short DNA was removed with circulomics short read eliminator kit. The remaining DNA was then library prepped for nanopore sequencing using the Genomic DNA by Ligation kit (SQK-LSK111) and loaded onto an R 10.34 flow cell. Nanopore reads were basecalled with guppy (version 6.2) and short (< 3000 bp) and low quality (Phred quality score < 10) reads were removed with nanofilt (6). Nanopore reads were assembled with Shasta (7), and contigs with one or more high quality (Expect value <= 10^{-10}) BLAST hits to previously published diazoplast genomes (NCBI accessions NZ_AP012549.1, NZ_AP018341.1) (8,9) were isolated to create a fragmented and incomplete draft assembly. Nanopore and illumina reads were mapped to the draft assembly with minimap2 (10) and BWA-MEM (11) and mapped reads were re-assembled with unicycler (12) to produce a high-quality circular diazoplast genome. Genome quality was assessed with Merqury and quast (13,14). Gene annotation of the diazoplast was performed with NCBI Prokaryotic Genome Annotation Pipeline. The complete sequence for the diazoplast of *E. clementina* is publicly available on NCBI (Accession: PRJNA944260, ID: 944260, assembly GCA_029919255.1).

Phylogenetic analysis

Using the corresponding *Phaeodactylum tricornutum* or *C. subtropica* (*Cyanothece* sp. ATCC 51142) gene sequences as queries, sequences of *Epithemia clementina* host genes for *psbC, rbcL* and 18S-rRNA and endosymbiont genes for *nifH* and 16S-rRNA were extracted from the Spades assembly by command-line BLAST (15,16). Sequence lengths extracted are as follows: 1391bp of *rbcL*, 1076bp of *psbC*, 1622bp of

18S-rRNA, 761bp of *nifH,* and 1410bp of 16S-rRNA. A nucleotide BLAST against the NCBI nr/nt database was performed to ensure correct identity of these sequences. Sequences used for phylogeny are gathered in Table S4. For each gene, sequences were aligned using MAFFT v7.490 (mafft-linsi –adjustdirectionaccurately –maxiterate 1000) (17). Gaps and highly variable regions in the alignment were removed with trimAl v1.2rev59 (18) using the gappyout flag and inspected by eye to ensure proper alignment and trimming. Concatenated alignments and data partitions were generated using SequenceMatrix v1.9 (19). Phylogenetic trees were inferred using IQ-TREE 2 (20) with ModelFinder (21) automatic model selection and node support tested with 2000 iterations of rapid phylogenetic bootstraps.

Metagenomic analysis and assembly

Metagenomic assembly was performed with flye (22) and contigs were polished 3x with racon (23). Eukaryotic contigs were removed with EukRep (24), and metagenomic binning was done with MetaBat2 (25). To investigate the possibility of nitrogen fixation by a free-living microbe, assembled contigs were searched against the UniProt reference proteomes database using Diamond (26) and results annotated as NifK (interpro IPR005976), NifD (interpro IPR005972), or NifH (interpro IPR005977) were isolated to identify putative nitrogen fixing microbes. To assess metagenomic sequence diversity, MAGs were classified using kraken2 (27) and metagenomic diversity was visualized with Krona (28).

Isotope labeling and NanoSIMS analysis

$^{15}N_2$-enriched Csi media was generated as follows. Bottles (250 mL) containing sterile Csi Media base were injected with 8 mL of $^{15}N_2$ gas (Cambridge Isotope Laboratories, Tewksbury, MA), vortexed at room temperature for 5 min, then stored for 24h at room temperature. $^{15}N_2$-enriched Csi media was then distributed into sterile serum vials. To verify the atom% enrichment of the prepared media, samples were sent for membrane inlet mass spectrometer analysis at the University of Hawaii at Manoa. The enrichment reached 47.5 ±1.8 atom%.

Pre-seeded 10mL vials with 2 days-old *E. clementina* cultures were filled with 2.5 mL of $^{15}N_2$-enriched Csi media. ^{13}C-bicarbonate ($NaH^{13}CO_3$, 50 µL at 0.5 M) was added to each sample. The vials were then filled to the top with non-enriched Csi media and sealed with no headspace. In parallel, control

samples were done with only non-enriched Csi media and non-labelled bicarbonate to assess natural abundance of ^{15}N and ^{13}C. Cultures were placed back in their culture conditions for either 12 hours of light or a full 24-hour cycle (12 h of light followed by 12 h of dark). At the end of the incubation period vials were emptied to leave only 3mL using a syringe and 1mL of 16% paraformaldehyde was added to the sample (final concentration 4%). Vials were immediately transferred to 4°C in the dark for 4 hours.

Embedding and sectioning was performed by the Cell Science Imaging Facility (Stanford, CA). Cells were pelleted and re-suspended in 10% Gelatin in 0.1M Sodium Cacodylate buffer pH 7.4 at 37°C and allowed to equilibrate 5 min. Cells were pelleted again, excess gelatin removed, then chilled in cold blocks and covered with cold fixative: 2% Glutaraldehyde and 4% paraformaldehyde in 0.1M Sodium Cacodylate pH 7.4. Left at 4°C overnight then changed to cold 1% Osmium tetroxide (EMS Cat# 19100) and allowed to warm to room temperature for 2 hours rotating in a hood. They were then washed 3 times with ultrafiltered water, then stained with 1% uranyl acetate for 2 hours at room temperature before being dehydrated in a series of ethanol washes for 20 minutes each beginning at 30%, 50%, 70%, 95%, changed to 100% ethanol 2X, then Propylene Oxide (PO) for 15 min. Samples were infiltrated with EMbed-812 resin (EMS Cat#14120) mixed 1:2, 1:1, and 2:1 with PO for 2 hrs each with leaving samples in 2:1 resin to PO overnight rotating at RT in the hood. The samples are then placed into EMbed-812 for 2 to 4 hours then placed into molds w/labels and fresh resin, orientated and placed into 65°C oven overnight. Embedded samples were sliced in semi-thin sections of 500 nM using a glass-knife mounted microtome.

Thin sections were placed on a silicon wafer and coated with gold and palladium. Samples were then loaded into a Cameca NanoSIMS 50L ion microprobe and were pumped down to ultra-high vacuum (10^{-9} Torr) for analysis. Ion imaging was conducted using a 16 keV Cs^+ primary ion beam (~5 pA) focused into ca. 100nm spot diameter (256 × 256 pixels, dwell time 1 ms per pixel). Seven electron multiplier detectors were set to simultaneously collect the masses of $^{12}C^-$, $^{13}C^-$, $^{12}C_2^-$, $^{12}C^{13}C^-$, $^{12}C^{14}N^-$, $^{12}C^{15}N^-$, and $^{32}S^-$ using a mass resolution M/DM > 9000. In addition, a secondary electron image was captured at each scan. A 50 µm^2 area containing the target cells was pre-sputtered for 2 min at ~70pA to remove any surface contamination prior to analysis. Analysis of 30 - 40 planes of the target cells was then done under a 30 µm2 raster. Quantification was done using Look@nanoSIMS software (29). The regions of interest were manually defined using signal from $^{32}S^-$ to separate three different fractions: the diazoplast (spherical form

and high $^{32}S^{-}$ and $^{12}C^{14}N$ signal), the host(N) (high $^{32}S^{-}$ and $^{12}C^{14}N$ signal), and the host(-N) (high $^{12}C^{-}$ and background $^{32}S^{-}$ and $^{12}C^{14}N$ signal). The manual outlining was done as best as possible but it is worth noting that some compartments with Host(-N) characteristics were too small to be properly outlined and were excluded if possible or included in Host(N) if present in the middle of that compartment. Representative examples of outlines are given in Fig. S7. Atom percent abundance was calculated as follows, for ^{15}N: $^{15}N^{12}C^{-}/[^{14}N^{12}C^{-} + {}^{15}N^{12}C^{-}]$ and for ^{13}C: $^{13}C^{12}C^{-}/[{}^{12}C_2^{-} + {}^{13}C^{12}C^{-}]$. Results for natural abundance are provided as average ± 2σ (95 percentile interval), and results greater than average natural abundance + 3σ were considered as enriched.

Isotopic ratio mass spectrometry (IRMS)

Isotopic labelling for IRMS was performed in T25 cell culture flasks. Flasks were seeded 3 days prior to the incubation with 10mL of culture. On the day of the experiment ^{13}C-bicarbonate or ^{12}C-bicarbonate ($NaH^{13}CO_3$, 50 µL at 0.5 M) was added to each sample and vented caps were swapped for sealed caps. After a 12h in light (day condition) or dark (night condition), cells were scraped off the flasks and harvested by centrifugation. Pellets were washed with fresh media containing no bicarbonate and transferred into aluminum cups. Samples were frozen and subsequently dried at 60°C. Samples were analyzed for $\delta^{13}C$, %C, %N, and C:N values by the Arizona Climate and Ecosystems (ACE) isotope laboratory at Northern Arizona University. Homogenized samples were weighed to 2.0 ± 0.1 mg in 4 x 6 mm tin capsules and processed by a Carlo Erba NC 2100 Elemental Analyzer (CE Instruments, Milan, Italy) connected to a Thermo-Finnigan Delta Plus XL (Thermo-Electron, Bremen, Germany) isotope ratio mass spectrometer.

Transmission Electron Microscopy (TEM)

Embedded samples used for nanoSIMS were also utilized for TEM imaging. Sections were taken approx. 80nm, picked up on formvar/Carbon coated 100 mesh Cu grids, stained for 30seconds in 3.5% Uranyl Acetate in 50% Acetone followed by staining in 0.2% Lead Citrate for 3 minutes. The sections were observed in the JEOL JEM-1400 120kV and photos were taken using a Gatan Orius 4k X 4k digital camera.

Nitrogenase activity assay

For acetylene reduction assays (ARAs), *E. clementina* cultures were scraped off the flasks and centrifuged (1,500xg, 4 min). They were washed once in fresh Csi-N media and resuspended in the appropriate media. From that culture, 2.3 mL was placed in an autoclaved 10 mL glass vial and sealed with a breathable sealing film. Note that the 10 mL vials have an actual volume of 12.3 mL, therefore the volume of culture was set to 2.3 mL to leave a 10 mL headspace in the vial. Vials were placed in their respective growth conditions for 3 days before the assay. For *C. subtropica*, fresh cultures were grown for 3 days in the desired conditions as described above and sampled for ARA at the time of the experiment.

Vials with 2.3mL of culture were sealed and injected with 1 mL of acetylene generated using calcium carbide and water. After one hour of incubation under culture conditions or desired conditions, the reaction was blocked by injection of 150 µL of 16% paraformaldehyde and kept at 4°C until further analysis. Ethylene, present in the 10 mL-head space, was quantified by injection of 1mL of the headspace into a gas chromatograph coupled with a flame ionization detector, Shimadzu GC-8A1F. A Porapak N 80/100 mesh 6' x 1/8" x 0.085" SSP/W column was used. The injector was set at 120°C and column at 80°C. Nitrogen was used as a carrier gas at 225kPa. Results were collected using a Shimadzu Chromatopac which performed automatic peak detection and quantification. A 1% ethylene standard gas was used to quantify the ethylene per signal detected. After gas analysis, cells were collected by centrifugation and chlorophyll *a* was extracted with 100% ethanol for quantification (30). Chlorophyll content serves as a proxy for the biomass in samples and was used to normalize the nitrogenase activity. Note that it only allows comparison between samples of the same species and not between species as cells differ in chlorophyll content. This method was more accurate than cell counting as *E. clementina* cells adhere to each other and surfaces.

Protein extraction and immunoblot

Cells were scraped and harvested at different time points of the day-night cycle by centrifugation at 3,000xg for 2 min. Pellets were deep-frozen in liquid nitrogen and stored at -80°C until protein extraction. Pellets were resuspended in 200µL of lysis buffer (10mM HEPES, 10mM EDTA, 0.5% Triton X-100, 2mM DTT). Cells were homogenized with a mix of 1mm and 0.5mM glass beads at 3000 strokes per minute for 2 minutes in a bead beater. Lysate was centrifuged at 15,000g for 5 min, 4°C and only supernatant was kept. Six volumes of cold acetone (-20°C) were added. After 1h at -20°C, proteins were pelleted at 15,000 g for

10 minutes, 4°C. Supernatant was kept for chlorophyll concentration measurement (30). Pellet was washed with 80% cold acetone, gently dried and resuspended at a final concentration of 0.1 µg/mL of chlorophyll which correspond to 1 µg/mL of total protein content in 1X lithium dodecyl sulfate loading buffer with 100 mM DTT. Samples were denatured at 70°C for 30 min, and vortexed periodically. Before loading, samples were pelleted at 15,000xg for 5 min. Precision Plus Protein™ All Blue Standards were used as a molecular ladder to assess protein molecular weight.

Proteins were separated by electrophoresis on NuPage Bis-Tris Gels, 4-12% polyacrylamide using MES buffer. They were then transferred using Bio-rad Trans-blot Turbo transfer onto a nitrocellulose membrane. Membranes were blocked in LiCOR blocking buffer (0.1% Casein, 0.2x PBS, 0.01% sodium azide) for 1 hour at room temperature. The FeMo nitrogenase (NifDK subunits) was immunoblotted with polyclonal goat-raised antibody (1:500 dilution) kindly provided by Dr. Dennis Dean from Virginia Tech, US. The large subunit of the hydrogenase (HupL) was immunoblotted with antibody (1:2500 dilution) raised in rabbit against amino acids 260 to 270 of HupL from *Anabaena* sp. PCC 7120 kindly provided by Dr. Paula Tamagnini from the Faculty of Sciences of University of Porto, Portugal. PsbA was used as an internal loading control; a 1:10,000 dilution was used of a rabbit antibody against the C-terminal of PsbA (AgriSera AB, Vanas, Sweden). Antibodies were diluted in 50% TBS-T / 50% LiCOR blocking buffer. After 2 hours with primary antibodies at room temperature, the membrane was washed 3 times with TBS-T and then incubated with LiCOR secondary antibodies (IRDye 800CW) for 1 hour (α-rabbit for PsbA and HupL and α-goat for NifDK). The membrane was rinsed twice with TBS-T and once with PBS and the blot was imaged using an infra-red LiCOR imager. Intensity of the signal was quantified using Image Studio Lite software v5.2. As NifDK and HupL have close similar weight, the nitrogenase was first immunoblotted and the membrane was stripped with 0.2M NaOH for 5 minutes, washed with water twice and the immunoblot was repeated for HupL.

Gene expression analysis

After 3 days of subculturing in the desired condition, cells were harvested at 1,500x*g* for 2 minutes, resuspended in Trizol Reagent (Invitrogen™, Carlsbad CA, USA) and then lysed by a combination of flash-freezing and sonication. mRNA was extracted using a Qiagen RNeasy® Plus Universal kit. mRNA

concentration was assessed by nanodrop. Following extraction, RT-qPCR was performed with NEB Luna® Universal One-Step RT-qPCR using 125ng of mRNA per 10µL reaction. Amplification was performed and monitored in a 96-well plate using a StepOnePlus™ Real-Time PCR system according to the instruction of the Luna kit and was followed by a melting curve. Primers used are detailed in Table S5. Specification of the amplification was validated first by PCR on gDNA and confirmed after the RT-qPCR of mRNA from analysis of the melting curves. DNA gyrase subunit B and 30S SSU ribosomal protein S1p were used as housekeeping genes. Expression was normalized to the weighted average of *gyrB* and 30S *ssu* expression.

Clark electrode measurements

Cultures of *C. subtropica* were subcultured 3 days prior to the assay, and supplemented or not with 1mM $(NH_4)_2HPO_4$. Samples at 4 hours into either the night or day period were directly transferred to the Clark electrode (Oxylab+ system, Hansatech instruments). Cultures were kept in suspension with a magnetic stirrer and exposed to light as needed using LEDs at 50 µmole photon m^{-2} s^{-1}. The rate of oxygen evolution was calculated from the variation of the oxygen concentration measured during a period of at least 5 minutes after stabilization of the measurement.

References

1. Adler S, Derch LM, Maier UG. Cultivation of the diatom Rhopalodia gibba. Endocytobiosis & Cell Research. 2010;(20).

2. Reddy KJ, Haskell JB, Sherman DM, Sherman LA. Unicellular, aerobic nitrogen-fixing cyanobacteria of the genus Cyanothece. J Bacteriol. 1993 Mar;175(5):1284–92.

3. Chen S, Zhou Y, Chen Y, Gu J. fastp: an ultra-fast all-in-one FASTQ preprocessor. Bioinformatics. 2018 Sep 1;34(17):i884–90.

4. Prjibelski A, Antipov D, Meleshko D, Lapidus A, Korobeynikov A. Using SPAdes De Novo Assembler. Current Protocols in Bioinformatics. 2020 Jun 1;70(1):e102.

5. Andrews S. FastQC: a quality control tool for high throughput sequence data [Internet]. Babraham Bioinformatics, Babraham Institute, Cambridge, United Kingdom; 2010. Available from: http://www.bioinformatics.babraham.ac.uk/projects/fastqc

6. De Coster W, D’Hert S, Schultz DT, Cruts M, Van Broeckhoven C. NanoPack: visualizing and processing long-read sequencing data. Bioinformatics. 2018 Aug 1;34(15):2666–9.

7. Shafin K, Pesout T, Lorig-Roach R, Haukness M, Olsen HE, Bosworth C, et al. Nanopore sequencing and the Shasta toolkit enable efficient de novo assembly of eleven human genomes. Nat Biotechnol. 2020 Sep;38(9):1044–53.

8. Nakayama T, Kamikawa R, Tanifuji G, Kashiyama Y, Ohkouchi N, Archibald JM, et al. Complete genome of a nonphotosynthetic cyanobacterium in a diatom reveals recent adaptations to an intracellular lifestyle. PNAS. 2014 Aug 5;111(31):11407–12.

9. Kneip C, Voβ C, Lockhart PJ, Maier UG. The cyanobacterial endosymbiont of the unicellular algae Rhopalodia gibba shows reductive genome evolution. BMC Evolutionary Biology. 2008 Jan 28;8(1):30.

10. Li H. Minimap2: pairwise alignment for nucleotide sequences. Bioinformatics. 2018 Sep 15;34(18):3094–100.

11. Li H. Aligning sequence reads, clone sequences and assembly contigs with BWA-MEM [Internet]. arXiv; 2013 [cited 2023 Mar 3]. Available from: http://arxiv.org/abs/1303.3997

12. Wick RR, Judd LM, Gorrie CL, Holt KE. Unicycler: Resolving bacterial genome assemblies from short and long sequencing reads. PLOS Computational Biology. 2017 Jun 8;13(6):e1005595.

13. Rhie A, Walenz BP, Koren S, Phillippy AM. Merqury: reference-free quality, completeness, and phasing assessment for genome assemblies. Genome Biology. 2020 Sep 14;21(1):245.

14. Gurevich A, Saveliev V, Vyahhi N, Tesler G. QUAST: quality assessment tool for genome assemblies. Bioinformatics. 2013 Apr 15;29(8):1072–5.

15. Altschul SF, Gish W, Miller W, Myers EW, Lipman DJ. Basic local alignment search tool. Journal of Molecular Biology. 1990 Oct 5;215(3):403–10.

16. Camacho C, Coulouris G, Avagyan V, Ma N, Papadopoulos J, Bealer K, et al. BLAST+: architecture and applications. BMC Bioinformatics. 2009 Dec 15;10(1):421.

17. Katoh K, Kuma K ichi, Toh H, Miyata T. MAFFT version 5: improvement in accuracy of multiple sequence alignment. Nucleic Acids Research. 2005 Jan 1;33(2):511–8.

18. Capella-Gutiérrez S, Silla-Martínez JM, Gabaldón T. trimAl: a tool for automated alignment trimming in large-scale phylogenetic analyses. Bioinformatics. 2009 Aug 1;25(15):1972–3.

19. Vaidya G, Lohman DJ, Meier R. SequenceMatrix: concatenation software for the fast assembly of multi-gene datasets with character set and codon information. Cladistics. 2011 Apr 1;27(2):171–80.

20. Minh BQ, Schmidt HA, Chernomor O, Schrempf D, Woodhams MD, von Haeseler A, et al. IQ-TREE 2: New Models and Efficient Methods for Phylogenetic Inference in the Genomic Era. Molecular Biology and Evolution. 2020 May 1;37(5):1530–4.

21. Kalyaanamoorthy S, Minh BQ, Wong TKF, von Haeseler A, Jermiin LS. ModelFinder: fast model selection for accurate phylogenetic estimates. Nature Methods. 2017 Jun 1;14(6):587–9.

22. Kolmogorov M, Bickhart DM, Behsaz B, Gurevich A, Rayko M, Shin SB, et al. metaFlye: scalable long-read metagenome assembly using repeat graphs. Nat Methods. 2020 Nov;17(11):1103–10.

23. Vaser R, Sović I, Nagarajan N, Šikić M. Fast and accurate de novo genome assembly from long uncorrected reads. Genome Res. 2017 May;27(5):737–46.

24. West PT, Probst AJ, Grigoriev IV, Thomas BC, Banfield JF. Genome-reconstruction for eukaryotes from complex natural microbial communities. Genome Res. 2018 Apr;28(4):569–80.

25. Kang DD, Li F, Kirton E, Thomas A, Egan R, An H, et al. MetaBAT 2: an adaptive binning algorithm for robust and efficient genome reconstruction from metagenome assemblies. PeerJ. 2019;7:e7359.

26. Buchfink B, Reuter K, Drost HG. Sensitive protein alignments at tree-of-life scale using DIAMOND. Nat Methods. 2021 Apr;18(4):366–8.

27. Wood DE, Lu J, Langmead B. Improved metagenomic analysis with Kraken 2. Genome Biol. 2019 Nov 28;20(1):257.

28. Ondov BD, Bergman NH, Phillippy AM. Interactive metagenomic visualization in a Web browser. BMC Bioinformatics. 2011 Sep 30;12(1):385.

29. Polerecky L, Adam B, Milucka J, Musat N, Vagner T, Kuypers MMM. Look@NanoSIMS – a tool for the analysis of nanoSIMS data in environmental microbiology. Environmental Microbiology. 2012;14(4):1009–23.

30. Ritchie RJ. Consistent Sets of Spectrophotometric Chlorophyll Equations for Acetone, Methanol and Ethanol Solvents. Photosynth Res. 2006 Jul 1;89(1):27–41.

Supplemental figures

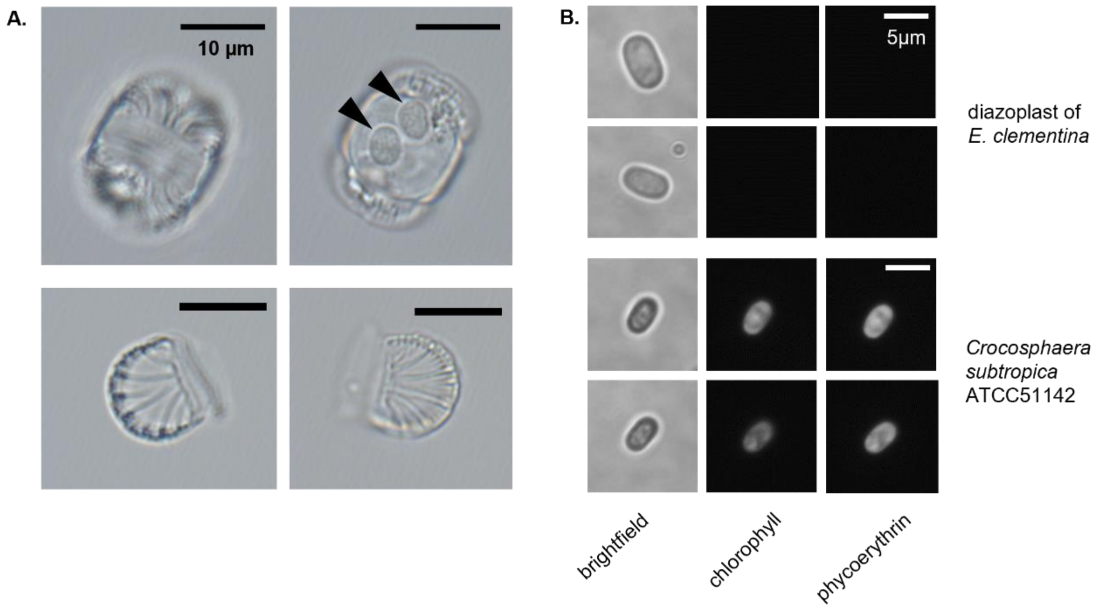

Figure S1. Supplemental micrographs of *E. clementina*.

(A) Light microscopy of *Epithemia clementina* testa with diazoplasts (black arrows) (B) Fluorescence microscopy of *E. clementina* diazoplast (top) and *C. subtropica* (bottom) showing fluorescence of chlorophyll and phycoerythrin.

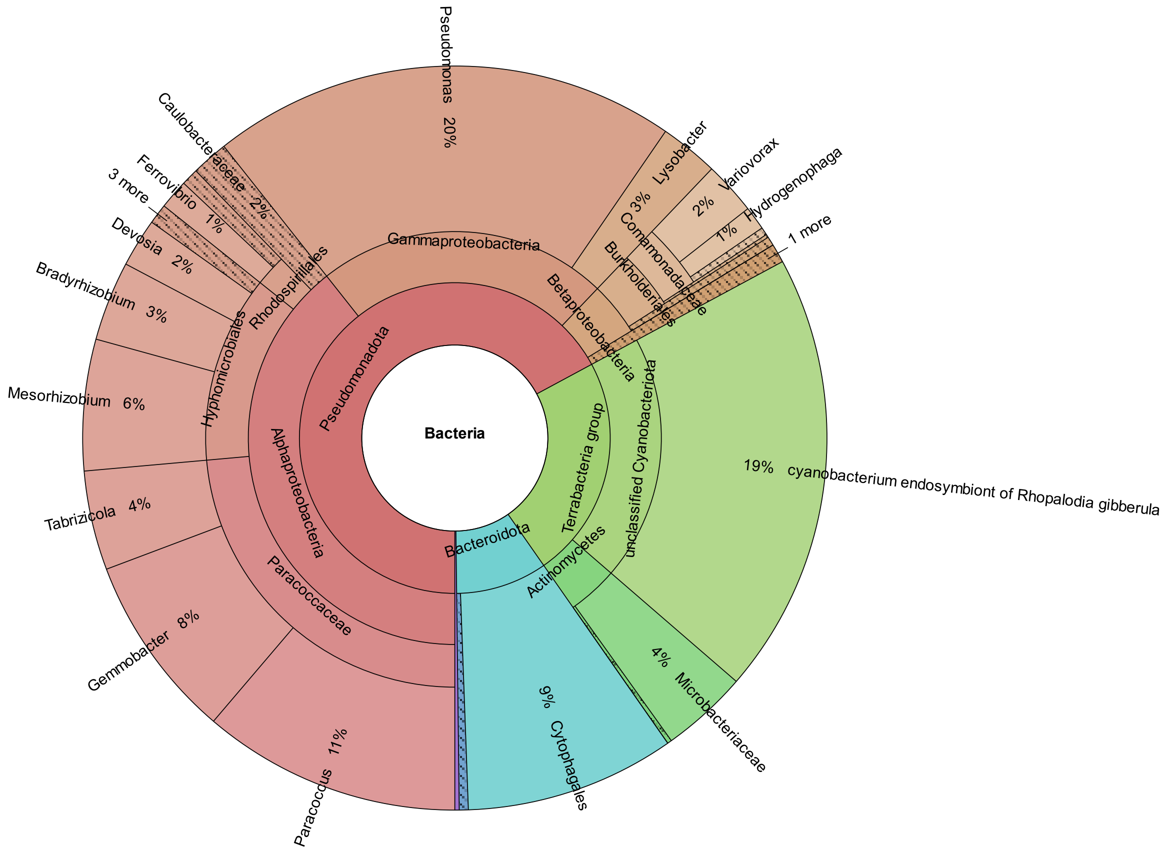

Figure S2. Diversity of the bacterial community associated with *E. clementina* cultures.

Krona visualization of kraken assignment of metagenome-assembled genomes. See Table S1 for abundance by MAGs.

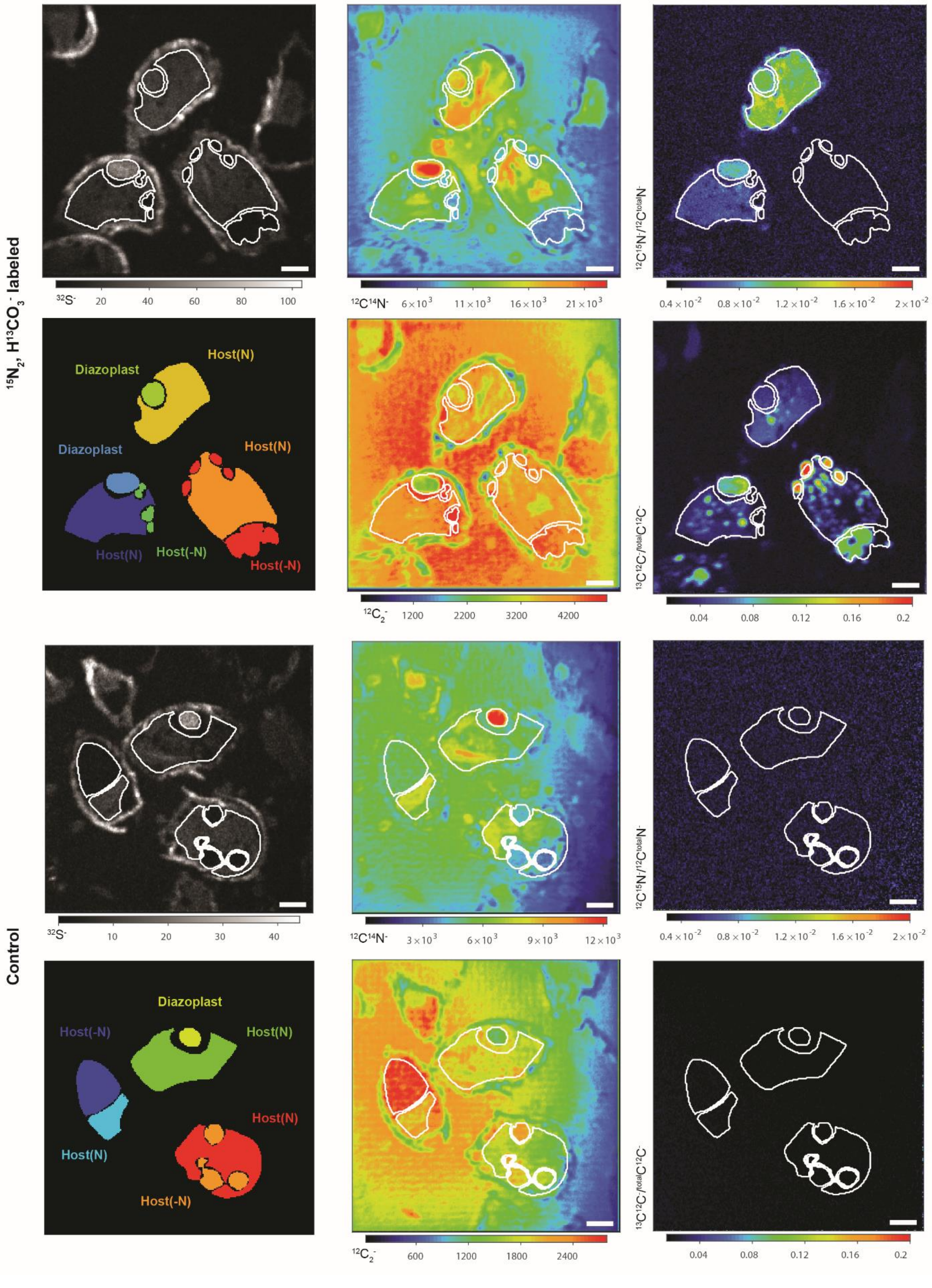

15N2, H13CO3- labeled
Control
32S-
20 40 60 80 100
12C14N-
6×10^3 11×10^3 16×10^3 21×10^3
12C15N-/12Ctotal N-
0.4×10^-2 0.8×10^-2 1.2×10^-2 1.6×10^-2 2×10^-2
Diazoplast
Host(N)
Host(-N)
12C2-
1200 2200 3200 4200
13C12C-/totalC12C-
0.04 0.08 0.12 0.16 0.2
32S-
10 20 30 40
12C14N-
3×10^3 6×10^3 9×10^3 12×10^3
12C2-
600 1200 1800 2400

Figure S3. Representative nanoSIMS images showing outlines of host(N), host(-N), and diazoplast compartments based on $^{32}S^-$ counts. The corresponding areas are shown in $^{12}C^{14}N^-$, $^{12}C_2^-$, $^{12}C^{15}N$ atom% and $^{12}C^{13}C$ atom% images.

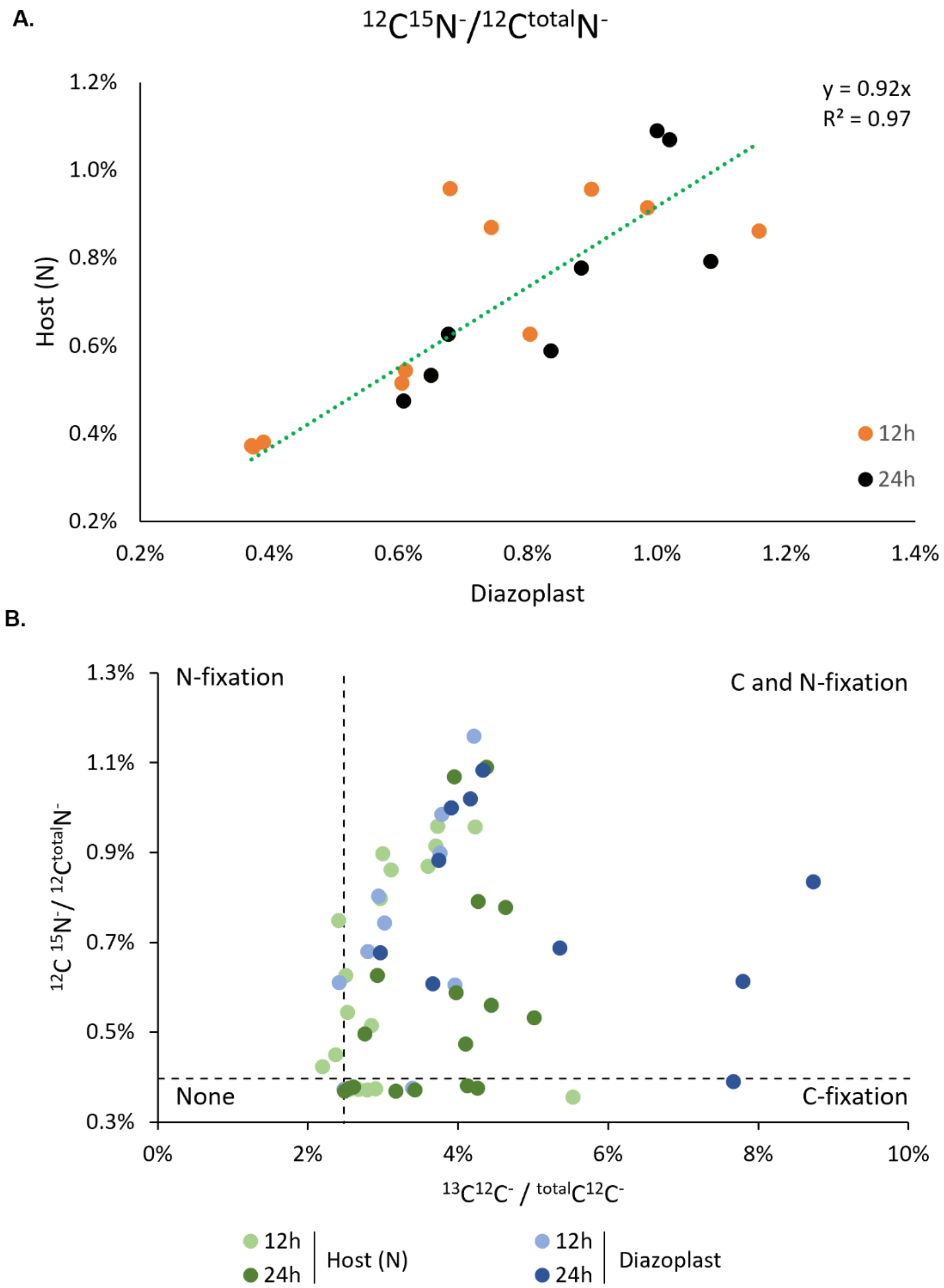

Figure S4. Carbon and nitrogen fixation in the *Epithemia* symbiosis.

(A) ^{15}N atom% in host(N) fraction compared to the corresponding ^{15}N atom% in diazoplast in individual cells. Linear regression is represented by the dashed line. Each dot is a single measurement. (B) ^{15}N atom% compared to the ^{13}C atom% for individual cells color-coded by compartment and incubation period. The dashed lines correspond to the upper 99.7% confidence interval observed from the mean of unlabeled control samples (average + 3*SD).

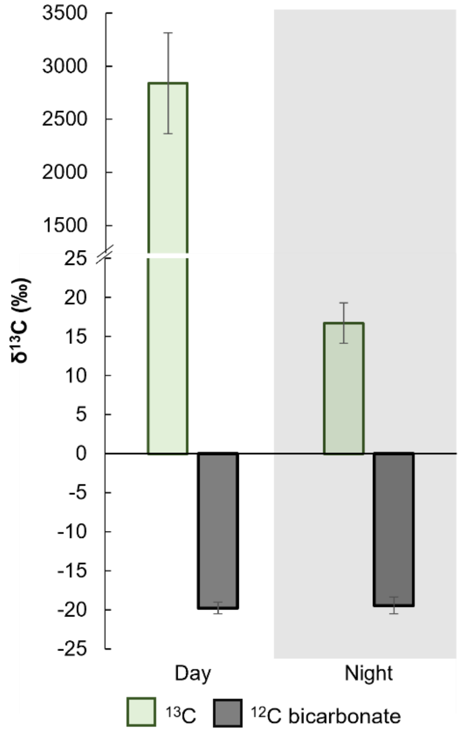

Figure S5: ^{13}C bicarbonate assimilation in *E. clementina*
IRMS quantification of δ13C (‰) after incubation with ^{13}C bicarbonate (green) or ^{12}C bicarbonate (black) during the day or the night period.

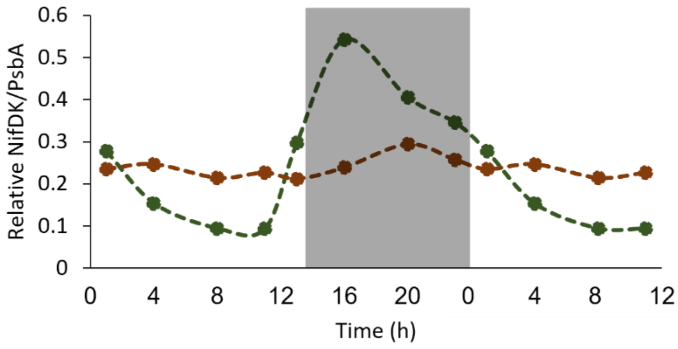

Figure S6. Quantification of NifDK to PsbA ratio from Fig. 3*B*. Timepoints (1, 4, 8 and 11h) were duplicated for better visualization of the cycle.

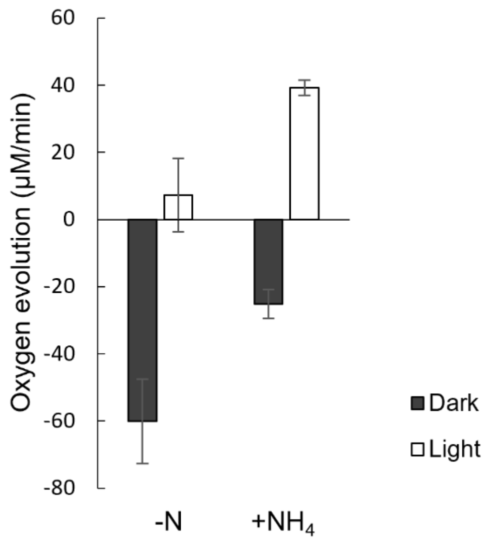

Figure S7. Respiration in *C. subtropica* is coupled to nitrogen fixation. Oxygen evolution measured by Clark electrode 4 hours into the night in *C. subtropica* cultured with or without ammonium. Mean value ± SD

Supplemental tables:

Table S1: MAGs analysis and taxonomic assignment.

Table S2: NCBI gene IDs. Genes present ID corresponding to protein involved in carbon metabolism, nitrogen metabolism, nitrogen regulation and circadian clock.

Table S3: Media recipe for Csi-N

	Stock concentration (M)	Final concentration (µM)	Volume in 1L of stock solution (mL)
$CaCl_2$	0.1	272	2.72
K_2HPO_4	0.1	57	0.57
β-Na_2 glycerophosphate*	0.1	163	1.63
$MgSO_4$	0.1	162.3	1.623
Na_2SiO_3 $9H_2O$	0.1	351.9	3.519
Hepes	1	10 mM	10
PIV Metals	-	-	3
B12 vitamin*	1 mg/mL	50 µg/L	0.05
Biotin*	2.5 mg/mL	50 µg/L	0.02
Thiamine HCl*	1 mg/mL	10 µg/L	0.01

PIV Metals:

	mg/100 mL	Concentration mM
Na_2EDTA $2H_2O$	100	2.69
$FeCl_3$ $6H_2O$	19.6	0.73
$MnCl_2$ $4H_2O$	3.6	0.18
$ZnCl_2$	1.04	0.076
$CoCl_2$ $6H_2O$	0.4	0.017
Na_2MoO_4 $2H_2O$	0.25	0.010

Media was adjusted to pH 7.5 and autoclaved for 20 minutes.

Solutions marked with (*) were filter-sterilized separately and added after autoclave.

Table S4: Source data for phylogeny

Table S5: List of primer used for RT-qPCR

Primer name	Primer sequence - Diazoplast	Primer sequence - *C. subtropica*
gyrB-For	GGTGGTAAATTCGGGGGAGG	TCG CTA TCA ACG GGC ATT AG
gyrB-Rev	TTCCCCTTTCGTAGCGTTGG	GCG CCC TCT CTC TAC TTT ATA C
30S-For	CACTCACGAGGATTTTGCGGC	CCT CCA GTC CAA CGA AAC AA
30S-Rev	CGTCTTCAGGATCGTCAACTCGG	GTT CCC AAG CTC GCA TAT ACT
NifK-For	CGTTGATCTGTTCCACCAGCC	CGG TGA TCC TGA TCT CGT AAT G
NifK-Rev	GTTGGCAAGCTTTAGCGGGG	CTT CAA ATT CCT TGG TGC TGT T
HupS-For	GAACCGGTTTGCTGGCCG	GGC CTG TTC TGG GAA TAC AA
HupS-Rev	GGTATTTAGCCCCTAAAGCTCCCCC	TAG GGA GGG ATG CCA TAA GA
glgA-For	CTCGTTTCGAACCTTGTGGC	CTT GGG AAG GAT TCC GCT TTA
glgA-Rev	CTCGTTTCGAACCTTGTGGC	ATT CGG CTG CTG ACT GAT AC
glgP-For	CGACCTAAGCCTCCGTTACC	GAC TCG GAA ACG GAG GAT TAG
glgP-Rev	TCGGCTGAATTCCTCATGGG	AAC TCG TAG CGG ATA CCA TAA C
zwf-For	GTTAACCCCTGGCTCTTCCC	GCC CAT CAA ACC AGT CCT AAT
zwf-Rev	GAACCTCCTAACGCTCTGGG	GTA GTT CTG ACC CTG GCA TTT
